# Triggered episodic vestibular syndrome and transient loss of consciousness due to a retrostyloidal vagal schwannoma: a case report

**DOI:** 10.3389/fneur.2023.1222697

**Published:** 2023-06-26

**Authors:** Maritta Spiegelberg, Ekin Ermiş, Andreas Raabe, Alexander Andrea Tarnutzer

**Affiliations:** ^1^Neurology, Cantonal Hospital of Baden, Baden, Switzerland; ^2^Department of Radiation Oncology, Bern University Hospital, University of Bern, Bern, Switzerland; ^3^Neurosurgery, Bern University Hospital, Bern, Switzerland; ^4^Faculty of Medicine, University of Zurich, Zurich, Switzerland

**Keywords:** case report, dysphagia, radiotherapy, transient loss of consciousness, dizziness

## Abstract

**Background:**

Various conditions may trigger episodic vertigo or dizziness, with positional changes being the most frequently identified condition. In this study, we describe a rare case of triggered episodic vestibular syndrome (EVS) accompanied by transient loss of consciousness (TLOC) linked to retrostyloidal vagal schwannoma.

**Case description:**

A 27-year woman with known vestibular migraine presented with a 19-month history of nausea, dysphagia, and odynophagia triggered by swallowing food and followed by recurrent TLOC. These symptoms occurred independently of her body position, resulting in a weight loss of 10 kg within 1 year and in an inability to work. An extensive cardiologic diagnostic work-up undertaken before she presented to the neurologic department was normal. On the fiberoptic endoscopic evaluation of swallowing, she showed a decreased sensitivity, a slight bulging of the right lateral pharyngeal wall, and a pathological pharyngeal squeeze maneuver without any further functional deficits. Quantitative vestibular testing revealed an intact peripheral-vestibular function, and electroencephalography was read as normal. On the brain MRI, a 16 x 15 x 12 mm lesion in the right retrostyloidal space suspicious of a vagal schwannoma was detected. Radiosurgery was preferred over surgical resection, as resection of tumors in the retrostyloid space bears the risk of intraoperative complications and may result in significant morbidity. A single radiosurgical procedure (stereotactic CyberKnife radiosurgery, 1 x 13Gy) accompanied by oral steroids was performed. On follow-up, a cessation of (pre)syncopes was noted 6 months after treatment. Only residual infrequent episodes of mild nausea were triggered by swallowing solid food remained. Brain MRI after 6 months demonstrated no progression of the lesion. In contrast, migraine headaches associated with dizziness remained frequent.

**Discussion:**

Distinguishing triggered and spontaneous EVS is important, and identifying specific triggers by structured history-taking is essential. Episodes being elicited by swallowing solid foods and accompanied by (near) TLOC should initiate a thorough search for vagal schwannoma, as symptoms are often disabling, and targeted treatment is available. In the case presented here, cessation of (pre)syncopes and significant reduction of nausea triggered by swallowing was noted with a 6-month delay, illustrating the advantages (no surgical complications) and disadvantages (delayed treatment response) of first-line radiotherapy in vagal schwannoma treatment.

## 1. Introduction

Vagal schwannomas are rare benign nerve tumors with a low incidence that hardly undergo malignant transformation and often present as slow-growing asymptomatic neck masses (1). Definitive pre-operative diagnosis and hence deciding on optimal treatment is often challenging, especially when located in the skull base within the retrostyloid space. In this study, we describe the rare case of a young female patient with recurrent episodes of dizziness, vertigo, and nausea accompanied by (near) transient loss of consciousness (TLOC) triggered by swallowing solid food, that was diagnosed with a retrostyloid vagal schwannoma and focus on the diagnostic workup, differential diagnoses, and treatment options.

## 2. Case description

**Figure 1:**
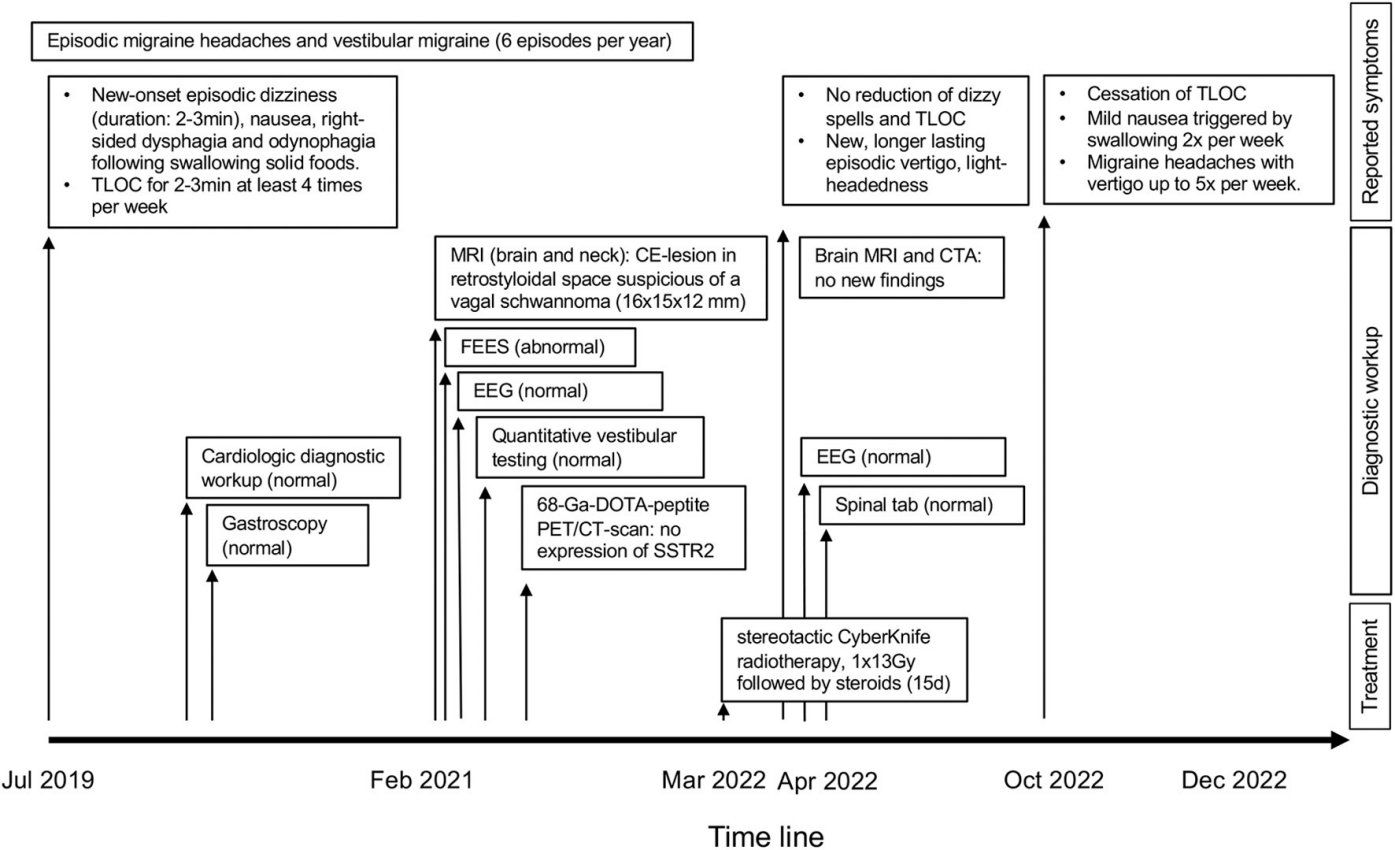
Illustration of the timeline of events, with reported complaints, diagnostic testing, and treatments is shown separately. CE, contrast-enhancing; CTA, computed tomography angiography EEG, electroencephalography; FEES, fiberoptic endoscopic evaluation of swallowing; Gy, gray; Ga-DOTA, gallium 68 labeled 1,4,7,10-tetraazacyclododecane-tetraacetic acid; MRI, magnetic resonance imaging; PET/CT, positron emission tomography/computed tomography; SSTR2, somatostatin receptor 2; TLOC, transient loss of consciousness.

A 27-year-old woman presented to our dizzy clinic in February 2021 with a 19-month history of recurrent episodes of dizziness (lasting 2–3 min) accompanied by nausea and dysphagia (located to the right side of the throat by the patient) and odynophagia following swallowing solid foods. Frequently, these episodes were followed by cold sweats, pallor, palpitations, and progressive right-sided tinnitus (with the tinnitus being known for more than 12 years). At the time of presentation, TLOC for approximately 2 to 3 min was noticed at least four times per week (see Figure 1 for the timeline of events). These symptoms occurred independently of her head and body position, and she could not avoid her episodes of TLOC with typical prevention maneuvers for vasovagal syncope such as squatting and leg crossing. Cervical pain, especially during horizontal head rotations or while lying on the right side, was denied. Due to these food-intake-related episodes, the patient lost 10 kg within 12 months. The patient's history was otherwise uneventful except for episodic migraine headaches without aura and a vestibular migraine (with episodes occurring about every other month and lasting for 1 day), and she did not take any medication on a regular basis. The patient's mother suffered from episodic headaches also, and an aunt had been diagnosed with a stroke.

Before presenting to our dizzy clinic, the patient had already received an extensive diagnostic work-up including a cardiologic assessment with no signs of structural heart disease or cardiac arrhythmia (with normal results on transthoracic echocardiography, stress echocardiography, long-term electrocardiography, and heart MRI). A gastroscopy showed normal results. On detailed neurologic and neuro-otologic examination at the time of presentation to our clinic, the patient showed no Horner syndrome, postural hypotension, or paroxysmal cough. Gait and balance testing was within normal range, and no signs of peripheral-vestibular loss-of-function were found on head-impulse testing. Hearing was normal. No symptoms of neurofibromatosis 1 could be detected.

With the episodes being triggered by swallowing solid foods, a fiberoptic endoscopic evaluation of swallowing (FEES) was performed. On FEES, a slight bulging of the right lateral pharyngeal wall with decreased sensitivity to touch was noticed. Pharyngeal squeeze maneuvers revealed a diminished contraction of the right lateral pharyngeal wall, which was considered pathological in comparison to the left side. Pharyngeal squeeze maneuvers also triggered cold sweats and dizziness without progression to syncope. No further functional deficits were noted on FEES, especially no recurrent laryngeal nerve paresis.

**Figure 2:**
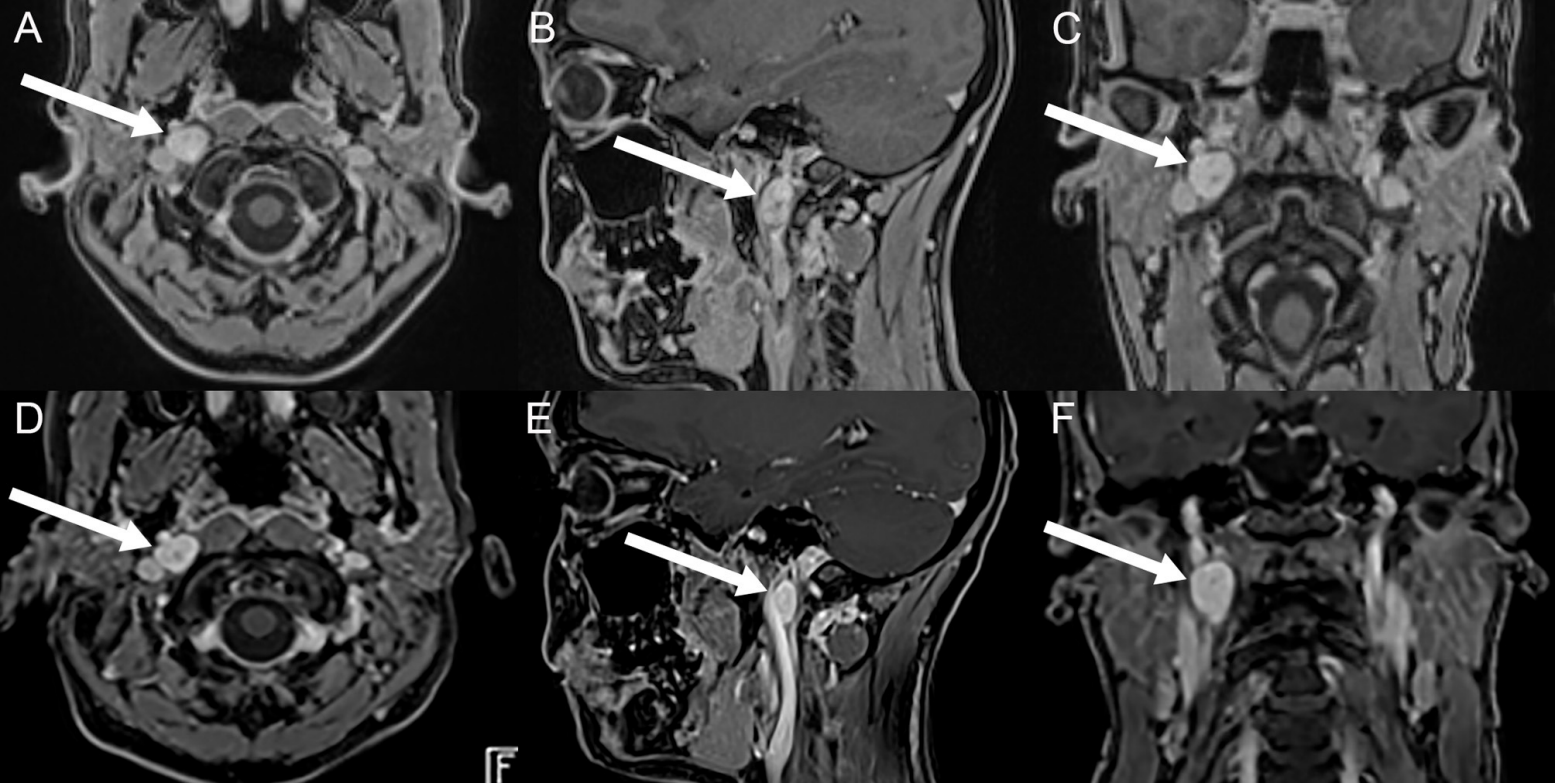
Illustration of the vagal schwannoma on brain MRI (axial, sagittal, and coronal post-contrast T1 volumetric interpolated breath-hold examination (VIBE) sequences) before treatment **(A–C)** and on follow-up, 6 months after treatment **(D–F)**. On initial imaging, a contrast-enhancing solid mass (marked with white arrows) in the right retrostyloid space in both the axial **(A)**, sagittal **(B)**, and coronal **(C)** plane can be seen. On follow-up, the solid mass is unchanged in size and morphology. Courtesy of MRI images **(A–C)**: Bilddiagnostik Basel, Switzerland.

Extensive neurologic evaluation including electroencephalography (EEG) and hemodynamic functional testing (Schellong test, 72 h-continuous electrocardiography, and 24 h blood pressure measurement), as well as quantitative vestibular testing (video head-impulse-testing (vHIT), cervical vestibular-evoked myogenic potentials (VEMPs), ocular VEMPs, and caloric irrigation), demonstrated normal results. On magnetic resonance imaging (MRI) of the brain a homogenous, contrast-enhancing lesion in the retrostyloidal space suspicious of a vagal schwannoma (dimensions = 16 x 15 x 12 mm) was detected (Figures 2A–C). On MRI of the cervical spine, no degenerative or post-infectious changes could be depicted. Subsequently, a 68-Ga-DOTA-peptite PET/CT scan was performed to better characterize the metabolic properties of this lesion. Testing showed no expression of SSTR2; hence, a paraganglioma or a glomus vagale tumor was considered unlikely.

Based on these findings, the case was presented to neuro-oncologists, neuro-radiologists, neurosurgeons, and otorhinolaryngologists at the interdisciplinary Schwannoma Board of the University Hospital of Bern, Switzerland. After evaluation, the board proposed a single radiosurgical procedure (stereotactic CyberKnife radiotherapy, 1x13Gy) accompanied by cortisone treatment (dexamethasone 4 mg per day, halving the dose every 3 days) for 10 days in order to cope with any radiation-induced edema. The patient agreed to this treatment, which was performed in March 2022 and was tolerated without any side effects. On 6 weeks of follow-up after treatment, the patient reported no reduction of the triggered episodes of (near) TLOC, dizzy spells, and exacerbated right-sided tinnitus. Weight was stable (60.7 kg). At the same time, however, longer-lasting episodes of vertigo and light-headedness were noticed, accompanied by fear of syncope, exhaustion, and transient left-sided hemisyndrome, which were interpreted as secondary functional symptoms/panic attacks due to complete restitution without treatment. Diagnostic procedures revealed a normal MRI scan of the neurocranium, a normal CT-angiogram, and EEG as well as normal cerebrospinal fluid (CSF) puncture lab and vasculitis screening lab results.

On further follow-up, the patient reported a cessation of episodes with (near) TLOC and dysphagia by October 2022, with residual infrequent episodes of mild nausea triggered by swallowing about twice per week. In contrast, migraine headaches associated with dizziness remained frequent (up to five episodes per week). On repeated brain MRIs obtained in September 2022, the vagal schwannoma was unchanged in size and morphology (Figures 2D–F). On the last follow-up in January 2023, the patient confirmed a regression of her migraine headaches accompanied by dizziness by approximately 50% of episodes and a continuing cessation of episodes with (near) TLOC.

Written informed consent was obtained by the patient for publication. Being a description of a single, clinical case, no ethics approval was required.

## 3. Discussion

Based on her clinical presentation with recurrent triggered dizziness (2), this patient met the diagnostic criteria for a triggered episodic vestibular syndrome (tEVS) (3). While most frequently tEVS is linked to positional changes in the context of benign paroxysmal positional vertigo (BPPV) and orthostatic hypotension, visual stimuli, or head motion (in patients with chronic vestibular loss-of-function) (3), other less known causes need to be considered as well. In this context, a structured history-taking addressing the specific type of trigger(s) is essential. With swallowing solid foods as a characteristic trigger, it is important to search for rare pathologies including parapharyngeal space-occupying lesions. In our patient, the diagnosis was based on the characteristic clinical presentation and the location of the tumor on MR imaging in the retrostyloidal parapharyngeal space. Importantly, these triggered episodes need to be distinguished from the patient's known migraine headaches and the vestibular migraine.

In most of these patients, neurogenic tumors which hardly undergo malignant transformation can be diagnosed (1, 4, 5). Nevertheless, they represent a considerable diagnostic and treatment challenge: the differentiation between a prestyloid lesion and a retrostyloid lesion is important due to the fact that the majority of retrostyloid parapharyngeal lesions are peripheral nerve sheath tumors, paragangliomas, or lymph node metastases (4). In contrast, prestyloid lesions are benign in 70–80 % and mostly of salivary (pleomorphic adenomas) or rarely neurogenic origin (trigeminal schwannomas). The diagnostic approach to retrostyloid masses includes MR-imaging as well as duplex ultrasound (6) and—after the exclusion of head and neck paragangliomas (7, 8)—transoral or transcervical fine-needle aspiration cytology (9). Vagus schwannomas are well-encapsulated tumors that most often are diagnosed between the third and sixth decade of life, presenting with a cervical, slow-growing mass. When palpable, characteristic findings of parapharyngeal neurogenic tumors can be found, specifically, this mass is typically mobile transversely, but not vertically, and dislocates the carotid artery and the jugular vein anteriorly (10). Paroxysmal cough and hoarseness can be initial symptoms. Schwannomas are often located in the retrostyloid parapharyngeal space but can even extend into the posterior cranial fossa via the jugular foramen (11).

### 3.1. Treatment options for vagal schwannoma

Different therapeutic approaches need to be evaluated and discussed, ranging from watchful waiting to radiotherapy and microsurgical resection. Since many vagal schwannomas are asymptomatic when diagnosed, observation with serial imaging (“watchful waiting”) may be a viable option depending on the size and location of the tumor as well as the age and overall health status of the patient (12). Little is known about the natural history of vagal schwannoma. As summarized by Sandler et al., most authors estimate a growth rate of 1–3 mm per year based on limited case series, anecdotal evidence, and extrapolation from the vestibular schwannoma literature (12). However, most patients on a watchful waiting strategy will eventually need targeted treatment either because of observed tumor growth or the occurrence of clinical symptoms, as in the case presented here.

Surgical excision has been considered the primary treatment option, being highly effective, however, depending on the characteristics of the tumor (location, size, and surgeon's experience) (12). Reports using intraoperative nerve monitoring have shown improved nerve preservation (12, 13). Reports on radiosurgery for vagal schwannomas are still limited (12, 14–16). Radiation therapy is a commonly employed primary treatment option for vestibular schwannomas and has been shown to be extremely effective in halting tumor growth in more than 90% of cases (17). Recently, radiosurgery has been used as a primary treatment for medium to small schwannomas and as a secondary treatment for residual or recurrent disease after microsurgery (15). On the one hand, vagal schwannomas (especially cervical vagal schwannomas) are much easier to access surgically than vestibular schwannoma and thus microsurgical removal while leaving the vagal nerve intact is feasible (12). On the other hand, surgical resection of tumors in the retrostyloid space bears the risk of intraoperative complications (including vagal nerve damage and vocal cord palsy, alterations in phonation and swallowing (including painful swallowing), emergence of a Horner syndrome, and dysfunction of the temporomandibular junction (18)) and may result in significant morbidity. Thus, radiosurgery could be considered in patients who have shown small (i.e., <30 mm in diameter) tumors with tumor growth as in our patient (from 16 mm to 20 mm diameter within 5 months) but are poor surgical candidates (18, 19). Importantly, the anticipated delay in treatment response of more than 6 months needs to be considered also when discussing different treatment options.

While the malignant transformation of vestibular schwannomas following therapeutic radiation has been reported in single cases (20), the estimated risk of a secondary malignancy or malignant transformation of a benign tumor in patients treated with radiosurgery remains low at long-term follow up [0.045% over 10 years (21)]. There are no data on the long-term outcome of vagal schwannoma after radiosurgery. For microsurgical approaches, data are very limited. Different surgical resection techniques seem to have similar recurrence rates (12, 22). Noteworthy, in a systematic review of vagal paragangliomas, the vagal nerve was functionally preserved in only 11 of 254 surgically treated patients (4.3%) (23).

Thus, with radiosurgery selected as a primary treatment strategy, the case reported here reflects a novel approach. Preference for radiosurgery over microsurgical resection was based on an acceptable low risk of neurological deterioration after treatment and the expected long-term local and functional control. To date, resection has been the mainstay treatment for patients with vagal schwannomas. Considering the difficulty in achieving tumor control by surgery alone and the high post-operative morbidity rate, radiosurgery may be an effective and safe modality and a reasonable alternative to surgical resection for small- to medium-sized schwannomas (16).

In our patient, follow-up including MRI scans 6 months after treatment demonstrated stable tumor size with no progression on imaging and a reported complete cessation of (pre)syncopes with residual infrequent (2x per week) episodes of mild nausea. The patient could return to work by November 2022 and reported no TLOC anymore. The frequency of her vestibular migraine headaches decreased, and she felt healthy again and got pregnant. Further 1 year of follow-up after stereotactic radiosurgery will enable us to further evaluate this treatment option in this special case.

## 4. Conclusion

Schwannomas arising from the vagal nerve are a rare, benign pathology usually resulting in unspecific symptoms. Definitive pre-operative diagnosis and hence deciding on optimal treatment is often challenging, especially when localized in the skull base and in the retrostyloid space. The differentiation between a prestyloid lesion and a retrostyloid lesion thus is crucial for differential diagnosis. Usually, surgical excision is required, especially in cervical schwannomas, but frequently, post-operative complications can affect patients lifelong, so surgical indications should be based carefully on the balance between risks and benefits (24). Primary radiosurgery may, therefore, present a valuable alternative approach, as demonstrated in our case. Importantly, a delay in symptoms resolving of at least 6 months must be taken into account when selecting this treatment strategy.

## Data availability statement

The original contributions presented in the study are included in the article/supplementary material, further inquiries can be directed to the corresponding author.

## Ethics statement

Ethical review and approval was not required for the study on human participants in accordance with the local legislation and institutional requirements. The patients/participants provided their written informed consent to participate in this study. Written informed consent was obtained from the individual(s) for the publication of any potentially identifiable images or data included in this article.

## Author contributions

MS: data collection, data analysis, interpretation of data for the work, drafting the manuscript, and revising the work critically for important intellectual content. AR: data analysis, interpretation, and revising the manuscript critically for important intellectual content. EE: data analysis, interpretation, and revising the manuscript critically for important intellectual content. AT: conception of the work, interpretation of data for the work, drafting the work, and revising it critically for important intellectual content. All authors approved the final version of the manuscript and agreed to be accountable for all aspects of the work in ensuring that questions related to the accuracy or integrity of any part of the work are appropriately investigated and resolved. The authors confirm that all persons designated as authors qualify for authorship, and all those who qualify for authorship are listed.
